# Supplementary material for: Protein deuteration via algal amino acids to circumvent proton back-exchange for 1H-detected solid-state NMR
Source: Chem Commun (Camb). Author manuscript; Available in PMC 2024 Sep 9. (PMC10928984; doi:10.1039/d4cc00213j)
Supplement: SI [file EMS198383-supplement-SI.pdf]

Electronic Supplementary Material (ESI) for ChemComm.
This journal is © The Royal Society of Chemistry 2024

Supporting Information for

Protein deuteration via algal amino acids to overcome proton back-exchange for ^{1}H-detected solid-state NMR of large proteins

By Hanna Aucharova[1], Alexander Klein[1], Sara Medina Gomez[1], Benedikt Söldner[1], Suresh K. Vasa[1], Rasmus Linser[1]*
[1] Department of Chemistry and Chemical Biology, TU Dortmund University, Otto-Hahn-Str. 4a, 44227 Dortmund, Germany.

Experimental procedures:

Protein production

Perdeuterated, u-$^{13}C/^{15}N$ minimal media were prepared according to standard protocols[1] on the basis of 2 g of $^{2}H_7$,$^{13}C_6$ -labeled glucose and 1 g $^{15}NH_4Cl$. Media for algal amino acid labeling were prepared by dissolving 10 g of ^{2}H, ^{15}N, ^{13}C-labeled ISOGRO® powder in a small volume of ultrapure water, followed by addition of 18 ml of 100 g/L K_2HPO_4 solution, 28 ml of 50 g/L KH_2PO_4 solution, 20 ml of 50 g/L $MgSO_4$ solution, and 0.3 ml of 37 g/L $CaCl_2$ solution. After addition of ultrapure water to 1 L, the pH of media was adjusted to 7.0 with NaOH, and the media were supplemented with 100 mg of ampicillin, and filter-sterilized using a 0.2 µm filter. Media for Inverse Fractional Deuterated (iFD) samples obtained according to Medeiros-Silva et al.[2]are H_2O-based standard M9 minimal media containing ^{15}N NH_4Cl and $^{13}C_6$,$^{2}D_7$-glucose.

Chicken α-spectrin SH3 domain samples were generated in three labelling schemes: A first sample was based on the iFD medium. A second sample with algal amino acids labelling was obtained from the ISOGRO®-based medium described above. In addition, a third SH3 sample was prepared using u-^{15}N, ^{13}C labeled M9 medium as a reference for quantification of sidechain protonation. In all cases, the proteins were expressed using *E. coli* BL21-DE3 cells grown in the respective growth media at 37˚C with induction of expression at an OD_{600} of ~0.8 using 1 mM IPTG. After induction, the cells were incubated overnight at 20˚C and harvested after 18 hours by centrifugation at 6000 x g at 4˚C for 20 min, and pellets were flash-frozen in liquid nitrogen and stored at -80˚C.

In all cases, SH3 protein for the solution NMR samples was purified using established chromatography methods.[3] Before lysis, the cell pellet was thawed at 4°C and resuspended in 1:5 volume of buffer A. To this mixture, 10 µg/mL DNase I and 1× Complete Protease Inhibitor Cocktail tablet were added and incubated at 4°C for 30 min. Cells were disrupted in a microfluidizer at 1.5 mbar in two rounds and centrifuged at 110,000 × g for 45 min. The supernatant was separated by decantation and diluted with buffer A (see Table S1 for buffer components) to approximately 80 % of the column volume (CV) of the anion exchange column. The solution was injected into the HiPrep QFF 16/10 column with a flow rate

of 3 ml/min, and unbound organic molecules were removed by washing with 2 CV of buffer A. The bound molecules were eluted by a linear gradient of 0-9% of buffer B (8 CV). The fractions were pooled and monitored by SDS-PAGE; the SH3-containing fraction was shifted to pH 3.5 using 3 M citric acid and concentrated to 10-12 mg/ml, avoiding concentrations higher than 14 mg/ml to prevent precipitation. The concentrate was further purified by size exclusion chromatography (SEC) using a Superdex 75 prep grade column with a volume of 120 ml. After injection with a flow rate of 1 ml/min, 0.5 CV was eluted before fractionation of 2 ml. The SH3-containing fractions were pooled and concentrated for NMR studies.

Table S1: SH3 purification buffers for Anion-Exchange and Size-Exclusion Chromatography and Dialysis.

**Buffer**	**Composition**
AEC Buffer A	20 mM Tris, pH 8.8 (adjusted with 30% $HCl_{aq.}$)
AEC Buffer B	20 mM Tris, 1 M NaCl, pH 8.5 (adjusted with 30% $HCl_{aq.}$)
SEC Buffer	20 mM citric acid, 150 mM NaCl, pH 3.5 (adjusted with 4M $NaOH_{aq.}$)
Dialysis buffer	H_2O, pH 3.5 (adjusted with 30% $HCl_{aq.}$)

Algal amino acid labelled and perdeuterated, u-^{13}C/^{15}N-labelled tryptophan synthase (TS) samples for solid-state NMR measurements were expressed using the above-described growth media upon induction via 0.4 mM IPTG at an OD_{600} of 0.6-0.8. TS expression and purification was performed according to the protocol described in Hilario et al.[4] *E.coli* cells were inoculated from a Petri dish using a pipette tip, and the culture was left to shake at 37 °C at 160 rpm until the OD_{600} reached 1.2. The culture was then cooled down on the bench for 30 min, after which protein expression was induced by adding 0.4 mM IPTG. The culture was incubated overnight at 30ºC upon shaking at 160 rpm. The cells were then harvested by centrifuging at 4,500 × g at 4 °C for 20 min, the supernatant was removed and the cells resuspended in cold T-buffer (5 ml of buffer/g cells, see Table S2 for buffer components). Cells were disrupted by two runs through the high-pressure homogenizer Emulsiflex C3. The cell lysate was centrifuged at 80,000 × g for 30 min at 4ºC, the supernatant was collected and the pellets discarded. T-buffer, supplemented with 30% (w/V) PEG8000, was added to the supernatant (0.25 ml/ml of supernatant), followed by addition of 0.5 M spermine (0.0125 ml/ml of supernatant). The sample was then transferred to centrifuge tubes, spun down for 10 min at 80,000 × g at 4ºC. The supernatant was collected and left at 4ºC on a stir plate for 48 h. After 24 h, T-buffer with 30% PEG8000 was added ($V_{\mathbf{30\%\ PEG\text{-}8000}}$ = $V_{\mathbf{Supernatant}}$ ÷ 23) and stirred for another 24 h. The protein solution was spun down at 80,000 × g for 30 min at 4ºC, the pellets were collected and washed in T-buffer containing 30% PEG8000 and 0.5 M spermine (3.2 ml of buffer per each gram of cells). After washing, the pellet was resuspended in B-buffer with pH 9 (4 ml/g of cells). The solution was then stirred at room temperature for 2 h until all protein dissolved and the insoluble fraction becomes white. The solution was then dialyzed twice against 2 L of B-buffer for 24 h at 4ºC. After that, the protein was dialyzed against P-buffer for 48 h at 4ºC, the buffer was changed once after 24 h. The protein precipitate was then collected, dissolved in Bicine buffer and dialyzed against bicine buffer three times. The final protein solution was concentrated to 15 mg/ml and used for micro-crystallization.

Microcrystalline samples of TS were prepared by diluting the protein solution 1:1 with crystallization buffer containing 3 mM *N*-(4′-trifluoromethoxybenzenesulfonyl)-2-aminoethyl phosphate (F9; an analogue of the natural α-site substrate 3-idole-d-glycerol-3′-phosphate (IGP)) as previously described.[5] Microcrystals were collected and washed with crystallization buffer, doped with ~10 mM Cu-EDTA and packed into a Bruker 1.3 mm magic-angle spinning (MAS) rotor.

Table S2: TrpS purification buffers

Buffer	Composition
T-Buffer	50 mM Tris-HCl, pH 7.8, 0.1 M NaCl, 1 mM DTT, 5 mM EDTA, 1 mM PMSF, 0.01 mM PLP
B-Buffer	50 mM Bicine pH 9 using CsOH, 1 mM EDTA, 0.01 mM PLP, 1 mM DTT
P-Buffer	0.1 M K_2HPO_4, 0.1 M KH_2PO_4, 5 mM EDTA, 0.01 mM PLP, 170 g/L $(NH_4)_2SO_4$, 1 mM DTT
Bicine buffer	50 mM Bicine, pH 7.8 (adjusted with CsOH)
Crystallization buffer	50 mM Cs-bicine buffer pH 7.8, 14 % PEG8000, 3.0 mM spermine

Solution NMR measurements

For all experiments, 2 mM SH3 samples, prepared as described above, in a 2 mM citrate buffer at pH 3.5 were used. Slight deviations in the protein concentration were factored in by a constant factor measured from the $^{1}H^{N}$ bulk signal from an HSQC experiment.

Solution 2D H(N)(CO)CA spectra using ^{2}H but no ^{1}H decoupling were recorded for the sample based on algal amino acid labeling as well as the iFD sample to assess the amount of H^{α} protonation. The principles of this strategy are described in more detail in Asami et al.[6] An additional 2D H(N)(CO)CA spectrum with ^{1}H decoupling was recorded for a fully protonated sample for reference and resonance assignment transfer. For the ^{1}H coupled spectra the Bruker pulse sequence *hncocagp2h3d* was modified by simply removing ^{1}H decoupling pulses. The 2D data sets were recorded using 128 scans and acquisition times of approximately 25 and 90 ms for ^{13}C and ^{1}H, respectively.

To assess the amount of protonation in all other aliphatic sites, additionally, ^{13}C constant-time HSQC spectra were recorded for all three SH3 samples, i. e., the ISOGRO, iFD, and fully protonated sample (for resonance assignment transfer and reference), whereas the spectra for the ISOGRO and iFD samples were acquired using ^{2}H decoupling. All spectra were recorded using 48 scans and the constant-time period set to $2/J_{CC}$, i.e., 26.6 ms. In all cases, the ^{1}H dimension was recorded for 82 ms, whereas approximately 25 ms were used again for the indirect ^{13}C dimension.

Solid-state NMR experiments

^{1}H, ^{13}C and ^{15}N triple-resonance experiments on microcrystalline TrpS were performed at 16.4 T (700 MHz ^{1}H Larmor frequency) on a Bruker NEO spectrometer equipped with a triple-resonance 1.3 mm MAS probe spinning at MAS rates of 55.555 kHz. The ^{1}H chemical shifts were referenced with respect to water, and ^{15}N and ^{13}C chemical shifts were referenced indirectly with respect to ^{1}H. Hard-pulse and CP parameters are summarized in Table S3 below. The pulse sequence for 3D-hCONH experiment is shown in Fig. S1. Each experiment was recorded with 2048 points in the direct dimension, 180 points in F2 (^{15}N), and 150 in F3 (^{13}C); number of scans being 8, acquisition time 50 ms in the ^{1}H dimension, 32 ms in the ^{15}N dimension, and 27 ms in the ^{13}C dimension. The total experimental time per experiment amounted to - 6 days. Line widths were measured using apodization with 50 Hz exponential line broadening, which value was then subtracted.

Table S3: experimental parameters

	F3 (^{1}H)	F2 (^{15}N)	F1 (^{13}C)	
**FID size**	2048	180	150	
**SW / ppm**	29.7545	39.1478	15.7749	
**Increment size / µs**		360	360	
**Aq. time / ms**	49.15	32.40	27	
**Scans**	16,	**d1**	1 sec	
**Hard pulses**	**µs**	**RF / kHz**		
^{1}H	1.65	151.5		
^{15}N	5	50		
^{13}C	3.63	68.87		
**H-CO CP**		**RF ^{1}H / kHz**	**RF ^{13}C / kHz**	**RF ^{15}N / kHz**
Contact time	1710 µs	24.46	38.46	-
Shape		100-50 tang	rectangular	-
**CO-N CP**		**RF ^{1}H / kHz**	**RF ^{13}C / kHz**	**RF ^{15}N / kHz**
Contact time	11000 µs	-	17.08	50
Shape		-	rectangular	90-100 ramp
**N-H CP**		**RF ^{1}H / kHz**	**RF ^{13}C / kHz**	**RF ^{15}N / kHz**
Contact time	162 µs	19.88	-	52.96
Shape		100-50 tang	-	rectangular
**C$^{\alpha}$ dec**		**RF ^{1}H / kHz**	**RF ^{13}C / kHz**	**RF ^{15}N / kHz**
Q3	300 µs	-	11.42	-

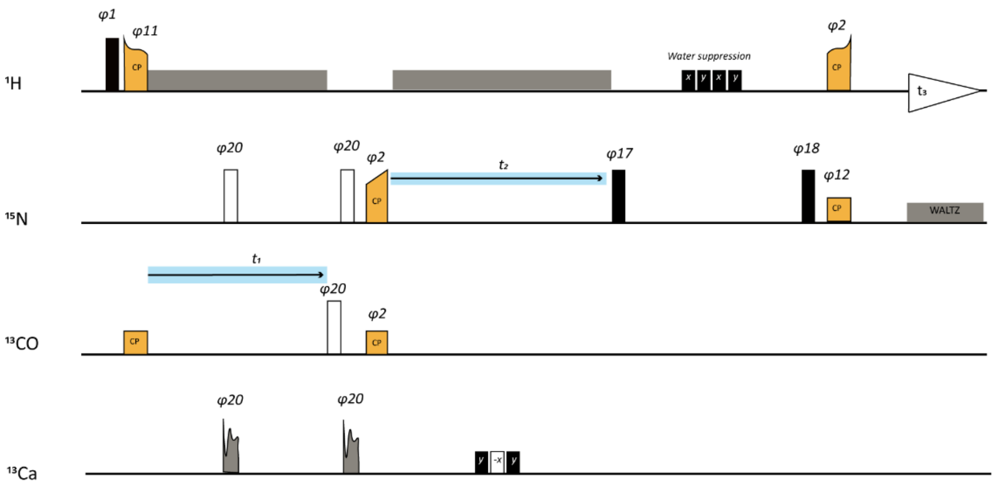

Fig. S1: Pulse sequence for the proton-detected hCONH experiment. Filled and empty rectangles and shapes indicate π/2 and π pulses, respectively. The ^{13}C inversion pulses were applied selectively as soft rectangular (on resonance) or Q3 shapes (off resonance). Orange boxes are spin-lock pulses for cross-polarization, and gray boxes indicate Waltz-16 decoupling (proton: 10 kHz, heteronuclear: 2 kHz). The four successive proton pulses are 10 kHz water dephasing pulses of 20 ms each. Phase cycling: ϕ1 = x, -x; ϕ2 = x; ϕ11 = x, x, x, x, -x, -x, -x, -x; ϕ12 = y; ϕ17 = y; ϕ18 = x, x, -x, -x; ϕ20 = y.

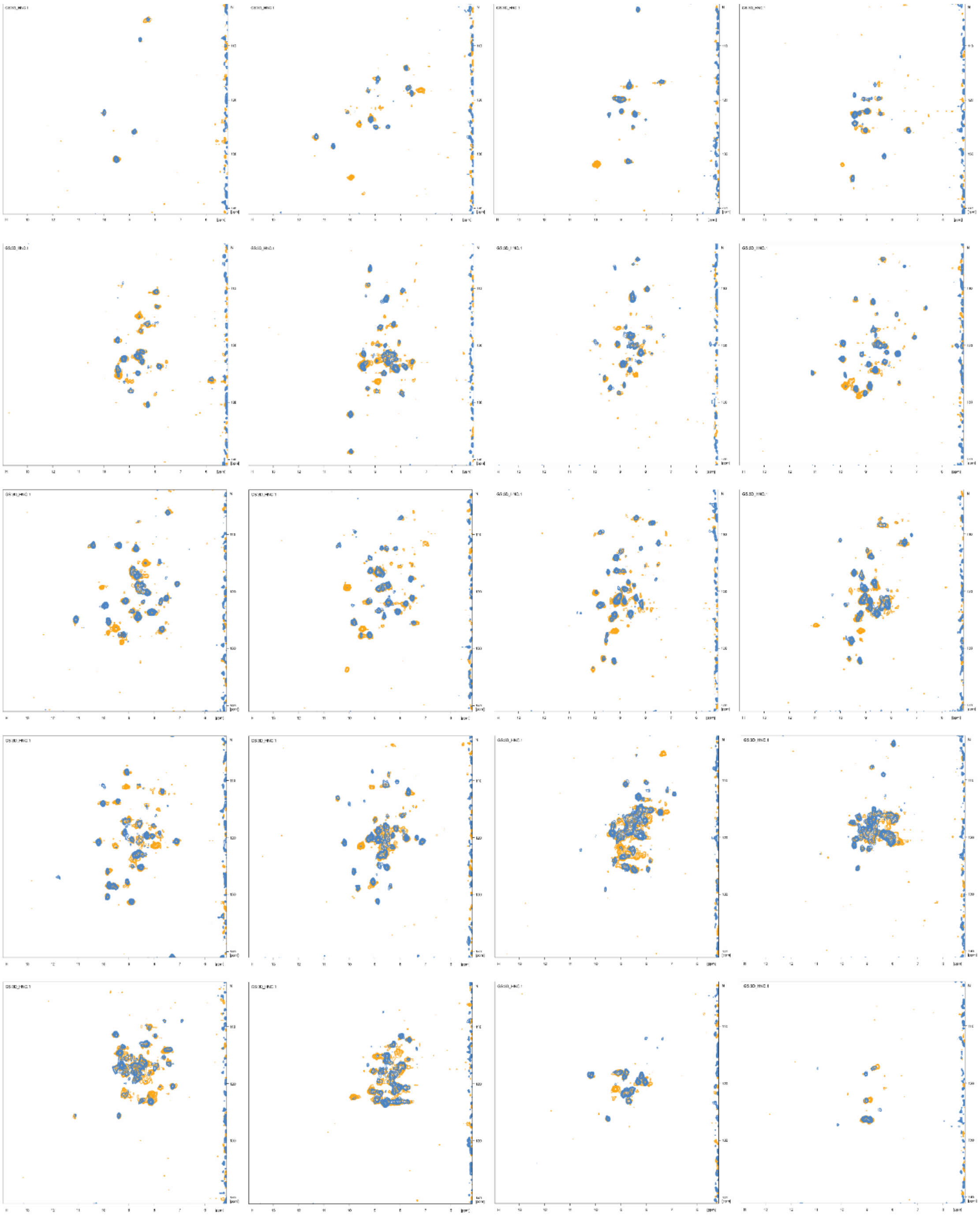

Fig. S2: Additional exemplary hCONH slices ($^{1}H/^{15}N$ planes) from perdeuterated (blue) and algal amino acid labeled samples (orange) in which the appearance of peaks with a poor proton back-exchange is witnessed.

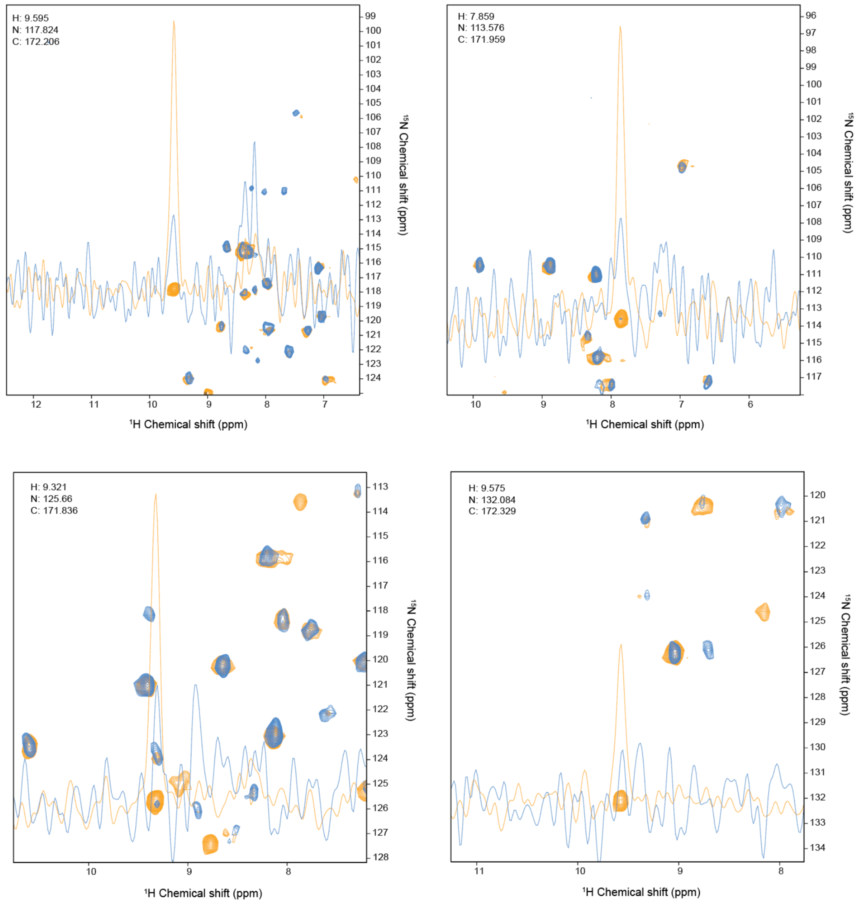

Fig. S3: Cross sections from representative slices through the 3D hCONH as in Main Text Fig. 2 and Fig. S2. Yellow and blue spectra correspond to algal amino acid labeled and perdeuterated/back-exchanged samples, respectively.

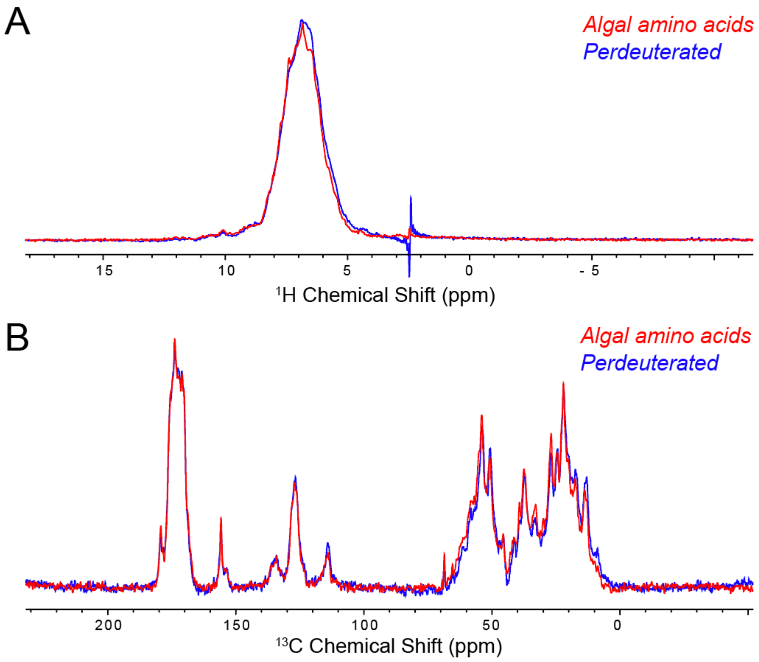

Fig. S4: hNH bulk spectra **(A)** and ^{13}C direct polarization spectra **(B)** for the perdeuterated/back-exchanged (blue) as well as the algal amino acid sample (red).

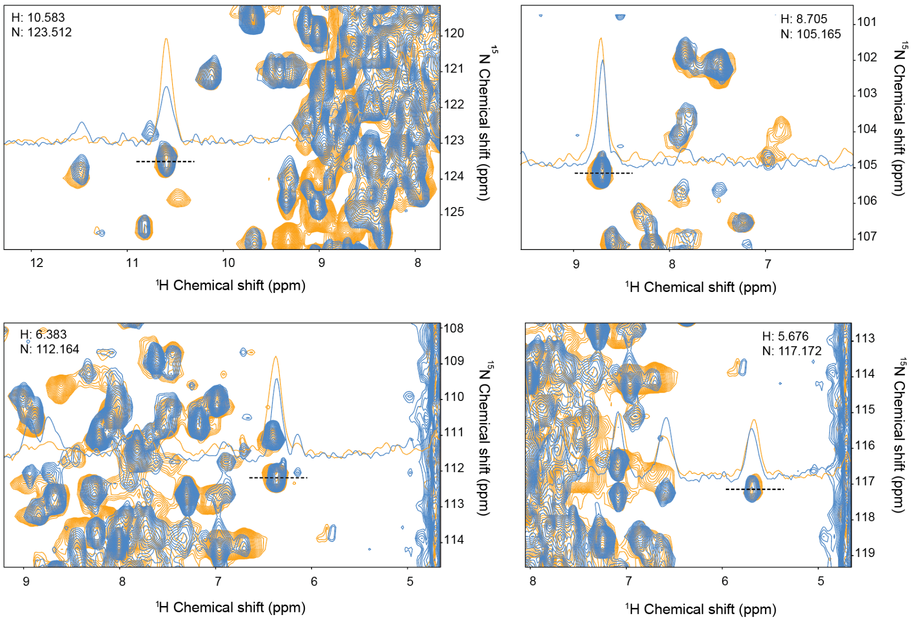

Fig. S5: Cross sections from Main Text Fig. 3.

Calculation of protonation levels in iFD and ISOGRO samples:

For all experiments, a 2 mM SH3 sample in a 2 mM citrate buffer at pH 3.5 was used. Slight deviations in the protein concentration were factored in by a constant factor measured from the $^1H^N$ bulk signal from an HSQC experiment.

The extent of protonation at the H^α position p_α was determined following the protocol of Asami et al.[6] However, here we directly considered the volumes of the 2D peaks as determined in CCPNmr V3[7], with V_H denoting the volume of the part of the CH doublet, which is not superimposed by the CD singlet, and $V_{H,D}$ respectively the overlapped peak of the multiplet consisting of the remaining CD singlet and the other half of the CH doublet.

$$p_\alpha = \frac{2V_H}{V_{H,D} + V_H}$$

The extent of protonation of all other (side-chain) protons was calculated as suggested in Asami et al.[6] A ^{13}C-CT HSQC was recorded for the iFD, the ISOGRO, and a fully protonated sample to obtain reference peak volumes for all side-chain resonances. For methyl groups, the two other isotopomers were considered as well with the respective relative amount of protons they carry, while for methylene groups only one additional isotopomer has to be considered.

$$c_{methyl} = p_{CH_3} + \frac{2}{3}p_{CH_2D} + \frac{1}{3}p_{CHD_2}$$

$$c_{methylene} = p_{CH_2} + \frac{1}{2}p_{CHD}\text{ , with}$$

$$p = \frac{V_i}{V_{i,{}^1H_{ref}}}$$

$V_{i,{}^1H_{ref}}$ is here the peak volume measured on the fully protonated reference sample. Only well-resolved and clearly identifiable resonances were taken into account. For easier visualization, side-chain protonation levels, and ultimately all protons, were averaged over the number of H^α and

methyl(ene) groups in the respective amino acid. Missing signals or splittings were interpreted as full deuteration (black arrows in Main tex Fig. 1), causing lower averaged overall protonation levels than averaged side-chain protonation levels.

Costs for perdeuterated/back-exchanged vs. algal amino acid based cultures in this work (labelled ingredients only):

Table S4: Approximate cost comparison for perdeuterated and algal-based media.

	**Perdeuteration (in Euro)**	**Algal labeling (in Euro)**
**D_2O (1 L)**	~400	
**$^2H_7$,$^{13}C_6$-glucose (2 g)**	~440	
**$^{15}NH_4Cl$ (1 g)**	~25	
**ISOGRO or similar (10 g*)**		~1400
total	*~865*	*~1400*

*The necessary amount was not systematically determined. Note that in contrary to the 10 g per liter used here (10 x the amount recommended for usage of ISOGRO as a supplement), Löhr et al. in their original work[8] only used a 1 x amount, which accordingly would make the algal labeling ~10 x cheaper than estimated here.

Literature:

[1] a) M. Cai, Y. Huang, R. Yang, R. Craigie, G. M. Clore, *J. Biomol. NMR* **2016**, *66*, 85-91; b) E. H. Anderson, *Proc. Natl. Acad. Sci. U. S. A.* **1946**, *32*, 120-128.

[2] J. Medeiros-Silva, D. Mance, M. Daniels, S. Jekhmane, K. Houben, M. Baldus, M. Weingarth, *Angew. Chem., Int. Ed.* **2016**, *55*, 13606–13610

[3] V. Chevelkov, K. Rehbein, A. Diel, B. Reif, *Angew. Chem., Int. Ed.* **2006**, *45*, 3878-3881.

[4] E. Hilario, L. Fan, L. J. Mueller, M. F. Dunn, *J. Vis. Exp.* **2020**.

[5] B. G. Caulkins, B. Bastin, C. Yang, T. J. Neubauer, R. P. Young, E. Hilario, Y.-m. M. Huang, C.-e. A. Chang, L. Fan, M. F. Dunn, M. J. Marsella, L. J. Mueller, *J. Am. Chem. Soc.* **2014**, *136*, 12824-12827.

[6] S. Asami, P. Schmieder, B. Reif, *J. Am. Chem. Soc.* **2010**, *132*, 15133-15135.

[7] S. P. Skinner, R. H. Fogh, W. Boucher, T. J. Ragan, L. G. Mureddu, G. W. Vuister, *J. Biomol. NMR* **2016**, *66*, 111-124.

[8] F. Löhr, V. Katsemi, J. Hartleib, U. Günther, H. Rüterjans, *J. Biomol. NMR* **2003**, *25*, 291-311.
